# Evaluation of the applicability of the Immuno-solid-phase allergen chip (ISAC) assay in atopic patients in Singapore

**DOI:** 10.1186/s13601-015-0053-z

**Published:** 2015-02-27

**Authors:** Amelia Santosa, Anand Kumar Andiappan, Olaf Rotzschke, Hung Chew Wong, Amanda Chang, Mei Bigliardi-Qi, De-Yun Wang, Paul Lorenz Bigliardi

**Affiliations:** Division of Rheumatology, University Medicine Cluster, National University Health System, Singapore, Level 10 Tower Block, 1E Kent Ridge Road, Singapore, 119228 Singapore; Yong Loo Lin School of Medicine, National University of Singapore, 10 Medical Drive, Singapore, 117597 Singapore; SIgN (Singapore Immunology Network), A*STAR (Agency for Science, Technology and Research), Singapore, Singapore; Department of Pharmacy, National University Health System, Singapore, Singapore; IMB (Institute of Medical Biology), A*STAR (Agency for Science, Technology and Research), Singapore, Singapore; Department of Otolaryngology, National University of Singapore, Singapore, Singapore

**Keywords:** ISAC, Specific IgE, Atopy, Sensitization, Skin prick test

## Abstract

**Background/Objective:**

Molecular-based allergy diagnostics are gaining popularity in clinical practice. Our aim was to evaluate their role in the tropics, given the inherent genetic and environmental differences.

**Methods:**

We recruited subjects with history of atopy and collected data on demographics and atopic symptoms using validated questionnaires. Subjects underwent a series of skin prick tests (SPT). Serum total and specific IgE levels were measured using ImmunoCAP FEIA and ImmunoCAP ISAC®, respectively. We describe their pattern of sensitization and agreement between test methods.

**Results:**

A total of 135 subjects were recruited; mean ± SD age of 31.18 ± 12.72 years, 52.7% female. Allergic rhinitis (AR) was the most prevalent clinical manifestation of atopy (70.7%), followed by atopic dermatitis (AD) (50.5%) and asthma (26.2%). Polysensitization was seen in 51.1% of subjects by both SPT and ISAC. House dust mites (HDM) were the dominant allergen, with sensitization in 67.8% and 62% of subjects on SPT and ISAC, respectively. A group of subjects with monosensitization to *B. tropicalis* was identified. HDM sensitization was strongly associated with AR, while AD and asthma were not associated with sensitization to any allergen. Agreement between SPT and ISAC was mostly suboptimal. Greatest agreement was documented for the measurement of HDM sensitization with both methods (κ = 0.64). Sensitization to the bulk of the remaining allergens in the ISAC panel was infrequent.

**Conclusion:**

Multiplex methods should not be used as a screening tool, especially in a population with lower rates of polysensitization and a dominant sensitizing allergen. There may be a role in adjusting the antigen spectrum in the ISAC panel to regional differences.

**Electronic supplementary material:**

The online version of this article (doi:10.1186/s13601-015-0053-z) contains supplementary material, which is available to authorized users.

## Introduction

Atopy is on the rise worldwide. Globalization has resulted in a steady increase in the prevalence of atopy in Asia to approximate that of Western countries [1,2]. This trend is influenced by the emergence of new risk factors for atopy related to urban living (such as pollution [2] and exposure to new food allergens), as well as the loss of factors which are perceived to be protective, like exposure to farming [3], endotoxins [4] and a wide array of microbes [5]. The impact of atopy is likely to be vastly different in tropical Asia when compared to temperate Europe and US, where most studies were performed. The applicability of in-vivo and in-vitro allergy tests in tropical environments deserves closer attention.

Molecular-based allergy diagnostics are rapidly gaining popularity in routine clinical care. With commercial availability of over 100 allergens, these offer the opportunity to assess sensitization to multiple allergens concurrently, and distinguish true sensitization from cross-reactivity [6]. The ImmunoCAP Immuno-solid-phase allergen chip (ISAC) (Thermo Fisher Scientific) is a fluorescent immunoassay platform, where allergens are immobilized on a microarray chip to allow simultaneous measurement of specific IgE antibodies to 112 components from 51 allergen sources [6]. Unlike the singleplex methods, ISAC gives only a semi-quantitative report of free, allergen specific IgE (sIgE) levels [7].

Studies of atopic patients in Asia documented a polysensitization rate of barely 30% [8,9]. House dust mites (HDM) were found to dominate the sensitization profile of Singapore pediatric and adult populations [10-12]. In comparison, European cohorts of atopic patients reported rates of polysensitization of up to 90% in their subjects [13], where concurrent screening of a large number of allergens could be advantageous [6]. The utility of multiplex platforms in populations with a more homogenous pattern of sensitization such as ours requires a closer cost-benefit analysis.

Our objectives were to: a) describe the pattern of sensitization in an atopic, symptomatic Singapore-Chinese adult patients with bronchial asthma, rhinitis and/or atopic dermatitis and identify possible new relevant allergens, b) compare the applicability of ISAC and skin prick test (SPT) in atopic patients and c) to evaluate the clinical utility of the ISAC multiplex assay in Asia, when compared to the traditional SPT.

## Methods

### Study population

Study subjects were Chinese patients seen for atopic symptoms (asthma, rhinoconjunctivitis and/or atopic dermatitis (AD)) at the internal medicine, allergy, dermatology or ENT (Ear, Nose, Throat) outpatient clinics at the National University Hospital, Singapore, who agreed to participate. Subjects were subjected to questionnaires, SPT and venipuncture. Additionally, we included data of healthy, non-atopic subjects from a locally conducted study which had previously obtained approval from the Institutional Review Board of the National University of Singapore (Singapore, IRB reference NUS 10–445) [12]. These individuals did not have any symptoms of atopy. Their demographics, SPT data and total serum IgE levels had been collected previously. For the current study, aliquots of their stored sera were used for the ISAC test. Written informed consent was obtained from all subjects. The conduct of our study was approved by the local Institutional Review Board (NHG ROAM:2011/02188).

### Terminology

In this study, atopy was defined by the presence of atopic symptoms (such as asthma, rhinitis, conjuncitivis and/or AD) plus either a positive SPT to at least one allergen and/or elevated total IgE in the presence of at least one positive specific IgE. As the population resides in a tropical environment we studied the presence of occult parasitic infections to rule out their influence on total serum IgE level through collaboration with the Swiss Tropical and Public Health Institute (Socinstrasse 57, P.O. Box, 4002 Basel, Switzerland). The presence of anti-parasite IgG against *Trichinella sp, Toxocara sp, Echinococcus granulosus, Fasciola hepatica, Schistosoma sp, Filaria sp and Strongyloides sp* was determined. Non-atopic controls were those who had none of the above-mentioned symptoms and had negative SPTs as well as negative specific IgE to all tested allergens. Atopic symptoms were based on clinical observation and/or patients’ history. A history of asthma was defined based on the European Community Respiratory Health Survey (ECRHS II) [14]. Allergic rhinitis (AR) was defined according to the Allergic Rhinitis and its Impact on Asthma (ARIA) document [15] and AD was diagnosed based on the GA2LEN definition [16].

### Skin prick test

SPT were conducted by trained allergy nurses or physicians and evaluated by a certified allergologist. All pricks were done on the volar aspect of the subjects’ forearm and read 15 minutes after application. We used histamine (1mg/ml) as our positive control, while physiological saline served as negative control (both from Allergopharma®). The SPT was considered to be positive if the wheal diameter was larger than 3mm. The SPT solutions used in our test panel were from Allergopharma® (A), Germany and Stallergenes® (S), France. They included: *Alternaria tenuis (A)*, *Cladosporium herbarum (A)*, *Aspergillus fumigatus (A)*, *Penicillium notatum (A)*, cockroach (A), grass mix (A, containing kentucky blue grass, meadow fescue, orchard grass, rye grass, timothy grass and velvet grass), tree mix I (A, containing alder, elm, hazel, poplar and willow) and II (A, containing birch, beech, oak and plane tree), grasses/cereals (A, containing grasses, barley, oat, rye and wheat), herbs (A, (containing *Artemisia vulgaris*, *Urtica dioica*, *Taraxacum vulgare*, *Plantago lanceolata*)), latex (A), *Dermatophagoides farinae (A), Dermatophagoides pteronyssinus (A)*, *Dermatophagoides farinae (S), Dermatophagoides pteronyssinus (S), Blomia tropicalis (S),* dog (S), cat (S), prawn (S), curry (S), coffee (S), wheat (S), soya (S) and pork (S). HDM sensitization on SPT was measured using allergen extracts from Allergopharma® and Stallergenes® to ensure the reproducibility of HDM allergens from different suppliers.

### Serum IgE

Total serum IgE levels were measured using Phadia® 100; results were reported in kIU/L and values above 25 kIU/L were considered to be elevated. Specific IgE levels were measured by ImmunoCAP ISAC®. Results were reported in ISAC Standardized Units (ISU) and categorized based on the manufacturer’s cutoff levels (<0.3 ISU, undetectable or very low; 0.3-0.9 ISU, low; 1–14.9 ISU, moderate/high; ≥15 ISU, very high) [17]. Values above 1 ISU were considered positive. In addition, we measured specific IgE by ImmunoCAP to tropomyosin (rDer p 10, Thermo Fisher Scientific) to confirm tropomyosin (Der p 10) results obtained by ISAC.

### Statistical analysis

All the statistical analyses were performed using IBM SPSS Statistics version 21 (SPSS Inc., Chicago, IL, USA). Descriptive statistics were used with median, minimum and maximum values to describe continuous variables; absolute number and percentage were reported for categorical variables. Logistic regression was performed to determine the association between sensitization and atopy status. Agreement was measured between allergens in the SPT and ISAC panels. Each allergenic source on the SPT was compared against the composite of the allergenic molecules within the ISAC panel that belong to the corresponding allergenic source, as well as to the individual allergenic molecules from the corresponding allergenic source. Cohen’s kappa statistics was used as a measure of agreement. The strength of agreement was as follows: Kappa < 0 was poor, 0–0.2 was slight, 0.21 to 0.4 was fair, 0.41-0.6 was moderate, 0.61-0.8 was good and 0.81-1 was excellent [18]. Statistical significance was set at p < 0.05.

## Results

ISAC was performed on 87 subjects. We report demographic and symptoms data for all recruited subjects (n = 135); statistics for analysis of agreement and association with atopic symptoms was performed on subjects with ISAC data (n = 87).

Of all recruited subjects, 71 (52.7%) were female with a mean age of 31.18 ± 12.72 years. The majority of subjects were born in Singapore (85.7%). AR was the most prevalent clinical manifestation of atopy, affecting 82/116 (70.7%) of subjects, followed by AD (49/97, 50.5%) and asthma (34/130, 26.2%) (Table 1). The mean total serum IgE was 133.83 ± 172.13 kIU/L (range 2–1018 kIU/L). Median total serum IgE was significantly higher in atopics (*vs* non-atopics) (85.80 kIU/L *vs* 17.60 kIU/L, p = 0.001), sensitized (*vs* non-sensitized) individuals (186.84 kIU/L *vs* 55.36 kIU/L, p = 0.004), HDM sensitized (*vs* non-HDM sensitized) individuals (193.24 kIU/L *vs* 55.95 kIU/L, p =0.005) and in those with (*vs* without) AR (110.50 kIU/L *vs* 35.20 kIU/L, p = 0.006). Levels did not differ between subjects with/without asthma and AD. Polysensitization was seen in 51.1% (68/134) of subjects by both SPT and ISAC.

**Table 1:** **Baseline characteristics**

**Subject characteristics**	**(n=135 unless otherwise specified)**
Gender (n = 131)	
Male	47.3%
Female	52.7%
Age (n = 131)	31.18 ± 12.72 years
Atopic status (n = 134)	
Atopic	92.6%
- Polysensitized	51.1%
- Monosensitized	17.2%
Non-atopic	7.4%
Atopic symptoms	
Allergic rhinitis	(82/116) 70.7%
Asthma	(34/130) 26.2%
Eczema	(49/97) 50.5%
Number of atopic symptoms	
One	38.5%
Two	43.6%
Three	11.5%

**Figure 1:**
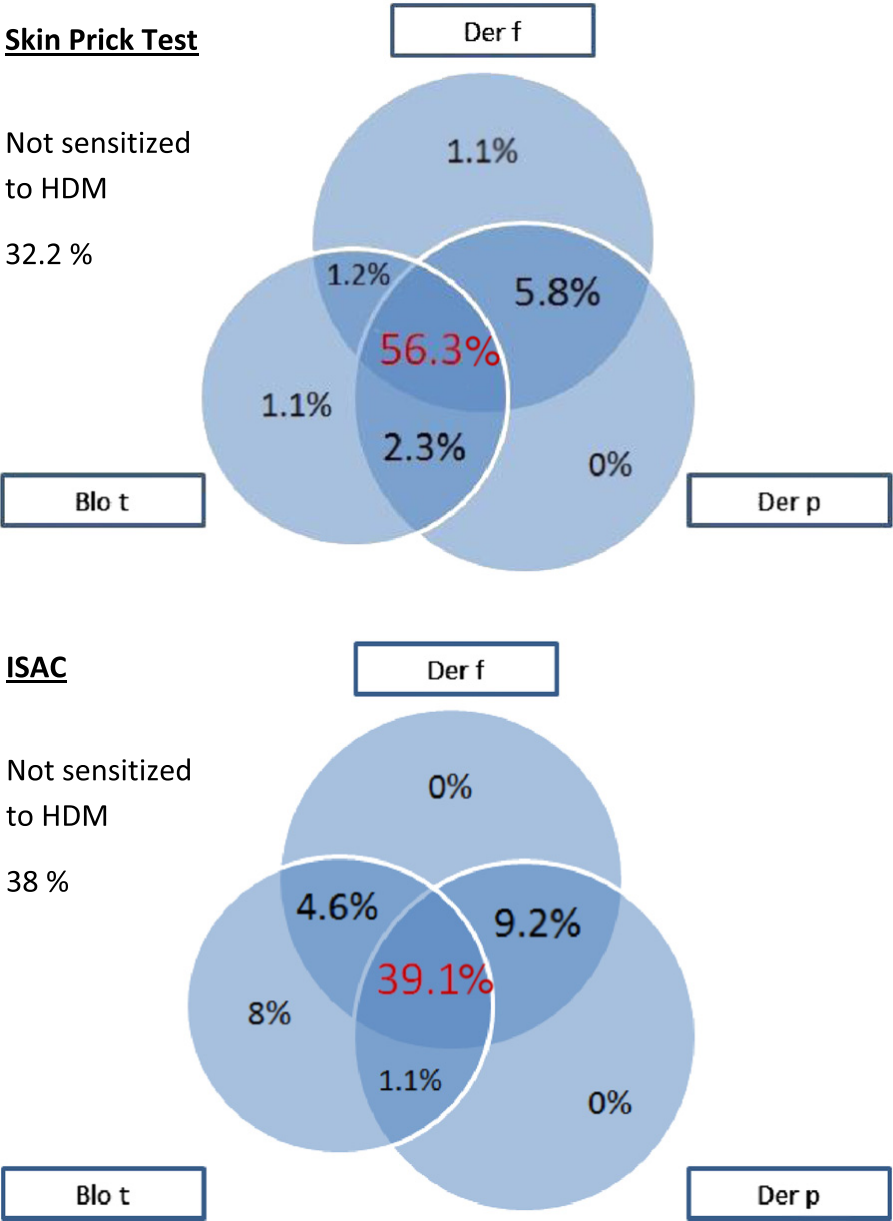
**HDM sensitization pattern on skin prick test and ISAC.**

In 87 subjects with ISAC data, further analysis was conducted. 59 (67.8%) were sensitized to HDM on SPT. Subjects with no HDM sensitization were less likely to be sensitized to any of the other allergens (OR (95%CI) 0.06 (0.01-0.15), p < 0.005) (data not shown). Monosensitization to HDM was present in 15 (17.2%). The majority (50.1%) had concurrent sensitization to a variety of other allergens in addition to HDM, while 25 (28.7%) did not have measureable sensitization to any allergen. Among individuals with HDM sensitization, one subject each was solely sensitized to *B. tropicalis* and *D. farinae*. Concurrent sensitization to all HDM species was seen in 49 (56.3%). In comparison, ISAC reported a lower proportion of HDM sensitized individuals (n = 54, 62%), but found that among them seven subjects (8%) had single-species sensitization to *B. tropicalis*, while none were solely sensitized to *D. farinae* or *D. pteronyssinus*. 34 (39.1%) were sensitized to all HDM species (Figure 1). While SPT reported that HDM sensitization was most frequently to *D. farinae*, ISAC found *B. tropicalis* to be the dominant HDM allergen.

HDM sensitization was significantly associated with AR on both SPT and ISAC. The odds ratio of AR in a subject sensitized to HDM on SPT was 10.92 (95% confidence interval 1.054–113.357, p = 0.044). On ISAC, every subject with sensitization to HDM had AR (p = 0.024). Sensitization was more frequently seen against HDM group 2 allergens. Agreement with SPT was numerically better with group 2 allergens (compared to group 1 mite allergens) (Table 2), and their association with AR symptoms was slightly stronger (OR(95%CI) 4.82 (1.88 to 12.34), p = 0.001 for AR in subjects sensitized to Der p 1 vs OR (95% CI) 8.08 (2.61 to 25.02), p < 0.001 for Der p 2 sensitized subjects; OR(95%CI) 5.33 (2.08 to 13.66), p < 0.001 for AR in subjects sensitized to Der f 1 vs OR (95% CI) 6.90 (2.43 to 19.61), p < 0.001 for Der f 2 sensitized subjects). We did not find any significant association between sensitization to any particular allergen with asthma or AD.

**Table 2:** **Agreement between skin prick test (SPT) and ISAC for house dust mites**

**SPT Allergen**	**ISAC allergen**	**Agreement Kappa (95%CI)**	**p**
*Dermatophagoides farinae*	Der f 1	0.46 (0.30-0.63)	<0.001
	Der f 2	0.55 (0.39-0.71)	<0.001
	Der f (total)	0.63 (0.46-0.79)	<0.001
*Dermatophagoides pteronyssinus*	Der p 1	0.43 (0.26-0.59)	<0.001
	Der p 2	0.49 (0.33-0.65)	<0.001
	Der p 10	0.04 (−0.05-0.08)	0.186
	Der p (total)	0.56 (0.40-0.73)	<0.001
*Blomia tropicalis*	Blo t 5	0.60 (0.44-0.77)	< 0.001

Please refer to Additional file 1: Table S1 for the remaining allergens.

The agreement between HDM SPT extracts from Allergopharma® and Stallergenes® was good (κ = 0.76, 95% CI 0.62-0.90). The overall agreement for HDM sensitization between SPT and ISAC was fair (κ = 0.57, 95%CI 0.40-74, p < 0.001), where agreement was greatest for *D. farinae* (κ = 0.63, 95% CI 0.46-0.79, p < 0.001). *D. pteronyssinus* and *B. tropicalis* followed with moderate levels of agreement (κ of 0.56 and 0.60, respectively) (Table 2).

Sensitization to allergens *other* than HDM was less frequent on both SPT and ISAC (Tables 3 and 4). The majority of subjects had owned a pet at some point of their lives (59.1%), most often dogs, rodents, birds and cats. However, pet ownership was not associated with measurable sensitization to the respective animals (both on skin prick and ISAC), regardless of whether pet ownership was current (ongoing exposure) or in the past. ISAC did not identify additional allergens which could be locally important from the large range of allergens included in their panel. The level of agreement between ISAC and SPT for allergens other than HDM was slight, or at most fair (Additional file 1: Table S1). Sensitization to HDM tropomyosin (Der p 10) was seen in 6.7% subjects by ISAC and in 5 (8.3% of 60 subjects) who had tropomyosin measurement by ImmunoCAP. There was moderate agreement between serum specific IgE against tropomyosin (Der p 10) by ImmunoCAP and ISAC (κ = 0.55, 95%CI 0.11-0.99, p < 0.001). Tropomyosin levels were significantly higher in subjects who were sensitized to HDM and cockroach, but not in those sensitized to shrimp.

**Table 3:** **Frequency of sensitization to the various allergens included in the skin prick test (SPT) panel**

**Allergen**	**SPT + (%)**
*Alternaria tenuis*	2.3
*Cladosporium herbarum*	2.3
*Aspergillus fumigatus*	2.3
*Penicillium notatum*	4.6
*Dermatophagoides farinae*	64.4
*Dermatophagoides pteronyssinus*	51.2
*Blomia tropicalis*	51.2
Cockroach	31
Grass mix	16.1
Cereals	10.3
Herbs	6.9
Tree mix I and II	12.6
Latex	4.6
Dog	28.7
Cat	21.8
Prawn	14.3
Curry	2.4
Coffee	2.4
Wheat	4.8
Soya	0
Pork	0

**Table 4:** **Proportion of sensitized subjects measured through the ISAC panel of 112 allergenic molecules**

**Allergen group**	**Allergen**	**Allergenic molecule**	**Very high (%)**	**Moderate/high (%)**	**Low (%)**	**Negative (%)**
Egg	Egg white	Gal d 1	0	0	0	100
		Gal d 2	0	0	0	100
		Gal d 3	0	0	2.2	97.8
	Egg yolk	Gal d 5	0	0	0	100
Milk	Cow’s Milk	Bos d 4	0	0	0.7	99.3
		Bos d 5	0	0	0	100
		Bos d 8	0	0	0	100
		Bos d lactoferin	0	0	0	100
Fish	Cod	Gad c 1	0	0	0	100
Shrimp	Shrimp	Pen m 2	0	1.5	0	98.5
		Pen m 4	0	0	0	100
Nuts & seeds	Cashewnut	Ana o 2	0	0	0.7	99.3
	Brazil Nut	Ber e 1	0	0	0	100
	Hazelnut	Cor a 9	0	0	0	100
	Walnut	Jug r 1	0	0	0	100
		Jug r 2	0	0	1.5	98.5
	Sesame	Ses i 1	0	0	0	100
Legume	Peanut	Ara h 1	0.7	0	0.7	98.5
		Ara h 2	0	0	0	100
		Ara h 3	0	0	0	100
		Ara h 6	0	0	0	100
	Soy	Gly m 5	0	0	0	100
		Gly m 6	0	0	0.7	100
Cereals	Buckwheat	Fag e 2	0	0	0	100
	Wheat	Tri a 14	0	0	0	100
		Tri a 19.0101	0	0	0	100
		Tri a aA_TI	0	0	0	100
Fruit	Kiwi	Act d 1	0	0	0.7	99.3
		Act d 5	0	0	0	100
Polcalcin	Birch	Bet v 4	0	0	0	100
	Timothy	Phl p 7	0	0	0	100
Grass pollen	Bermuda	Cyn d 1	0	1.5	6	92.5
	Timothy	Phl p 1	0.7	2.2	0.7	96.3
		Phl p 2	0	0.7	0	99.3
		Phl p 4	0	0	4.5	95.5
		Phl p 5	0.7	1.5	0	97.8
		Phl p 6	0	0.7	1.5	97.8
		Phl p 11	0	0	2.2	97.8
Tree pollen	Alder	Aln g 1	0	0	0.7	99.3
	Cedar	Cry j 1	0	0	1.5	98.5
	Cypress	Cup a 1	0	0	3	97
	Olive Pollen	Ole e 1	0	0	0	100
		Ole e 9	0	0	0	100
	Plane Tree	Pla a 1	0	0	0	100
		Pla a 2	0	0	1.5	98.5
Weed pollen	Ragweed	Amb a 1	0	0	0	100
	Mugwort	Art v 1	0	0	0	100
	Goosefoot	Che a 1	0	0.7	0.7	98.5
	Wall pellitory	Par j 2	0	0	1,5	98.5
	Plantain	Pla l 1	0	0	0.7	99.3
	Saltwort	Sal k 1	0	0	0	100
Animals	Cat	Fel d 1	1.5	5.2	0	93.3
		Fel d 4	0	2.2	0.7	97
	Dog	Can f 1	1.5	2.2	0.7	95.5
		Can f 2	0.7	0	0	99.3
		Can f 5	0	1.5	0.7	97.8
	Horse	Equ c 1	0	0.7	0.7	98.5
	Mouse	Mus m 1	0	1.5	0	98.5
Mould	Alternaria	Alt a 1	0	0.7	0	99.3
		Alt a 6	0	0	0.7	99.3
	Aspergillus	Asp f 1	0	0	0	100
		Asp f 3	0	0.7	0	99.3
		Asp f 6	1.5	0.7	0.7	97
	Cladosporium	Cla h 8	0	0	0	100
Mite	*Blomia tropicalis*	Blo t 5	12.7	39.6	3.7	44
	*D. farinae*	Der f 1	3	33.6	9	54.5
		Der f 2	17.9	20.9	2.2	59
	*D. pteronyssinus*	Der p 1	6.7	29.1	6.7	57.5
		Der p 2	11.9	23.1	3.7	64.9
	*Lepidoglyphus destructor*	Lep d 2	3.7	17.2	8.2	70.9
Insects	Cockroach	Bla g 1	0	0	0	100
		Bla g 2	0	0	0	100
		Bla g 5	0	0	0	100
Venom	Bee	Api m 1	0	0	1.5	98.5
		Api m 4	0	0	0.7	99.3
	Paper wasp	Pol d 5	0	0.7	2.2	97
	Common wasp	Ves v 5	0	0.7	1.5	98.8
Parasites	*Anisakis sp.*	Ani s 1	0	0	0	100
Latex	Latex	Hev b 1	0	0.7	0	99.3
		Hev b 3	0	0.7	0	99.3
		Hev b 5	0	0	0	100
		Hev b 6.01	0	0	0.7	99.3
Tropomyosin	*Anisakis sp.*	Ani s 3	0	0.7	1.5	97.8
	Cockroach	Bla g 7	0	0	1.5	98.5
	Mite	Der p 10	0	3	3.7	93.3
	Shrimp	Pen m 1	0	0	0	100
Serum albumin	Cow	Bos d 6	0	0	0	100
	Dog	Can f 3	0	0.7	0	99.3
	Horse	Equ c 3	0	0	0	100
	Cat	Fel d 2	0	0	0	100
nsLTP	Peanut	Ara h 9	0	0	0	100
	Hazelnut	Cor a 8	0	0	0	100
	Walnut	Jug r 3	0	0.7	0	99.3
	Peach	Pru p 3	0	0	0	100
	Mugwort	Art v 3	0	0	0	100
	Olive pollen	Ole e 7	0	0	0	100
	Plane tree	Pla a 3	0	0	0	100
PR-10 proteins	Birch	Bet v 1	0.7	0.7	0	98.5
	Hazel pollen	Cor a 1.0101	0	0.7	0.7	98.5
	Hazelnut	Cor a 1.0401	0	0.7	0.7	98.5
	Apple	Mal d 1	0	0	0.7	99.3
	Peach	Pru p 1	0	0	0	100
	Soybean	Gly m 4	0	0	0	100
	Peanut	Ara h 8	0	0	0	100
	Kiwi	Act d 8	0	0	0	100
	Celery	Api g 1	0	0	0	100
TLP	Kiwi	Act d 2	0	0	0	100
Profilin	Birch	Bet v 2	0	0	0.7	99.3
	Latex	Hev b 8	0	0.7	2.2	97
	Mercury	Mer a 1	0	0.7	1.5	97.8
	Timothy	Phl p 12	0	0	0.7	99.3
CCD	Bromelain	MUXF 3	0	0.7	0.7	98.5

≥15 ISU, very high ; 1–14.9 ISU, moderate/high; 0.3-0.9 ISU, low; <0.3 ISU negative.

Only one subject out of 60 for whom serum tests for parasite IgG were conducted had definite immunologic reactivity to parasite antigen (*Toxocara sp*) (results not shown). The subject was atopic with measurable sensitization to a variety of other tested allergens. The subject’s total serum IgE level was comparable to the other atopic individuals.

## Discussion

### Pattern of sensitization

The number of polysensitized individuals in our cohort is lower compared to Western populations [13] and slightly higher than in previously conducted Asian studies [8,9]. This is possibly due to the inclusion of a large range of allergens and because we targeted an adult, symptomatic population.

As shown in previous publications [10,12], HDM is the dominant allergen in Singapore. Expectedly, co-sensitization with *D. farinae* and *D. pteronyssinus* was frequent, consistent with evidence of significant homology between the two allergens [19]. The lower degree of structural similarity between *B. tropicalis* and the *Dermatophagoides* translated to less frequent co-sensitization between them [20]. It is noteworthy that a group of symptomatic individuals was monosensitized to *B. tropicalis* with both methods (1.1% SPT vs 8% ISAC). Such information is pertinent in patients planned for specific immunotherapy, since commercially available HDM immunotherapy kits are largely targeted against *D. farinae* and *D. pteronyssinus* antigens. Immunotherapy kits may need to be individualized to the patients’ sensitization profile.

Only sensitization to HDM was associated with AR. No allergen was independently associated with asthma or AD, although this could be due to the smaller number of patients in each of these groups. Interestingly, although HDM sensitization on ISAC was documented less frequently than on SPT, it predicted AR in all subjects it identified, suggesting perhaps that while SPT has better sensitivity, ISAC may be more specific for clinical symptoms. We detected occasional sensitization to a variety of other allergens but none were associated with symptoms of atopy. Therefore, in a population with a well-defined dominant allergen, routine clinical screening with a multiplex assay is not likely to value-add to a patient’s management and may in fact introduce the danger of erroneous avoidance of multiple allergens. Careful interpretation of the data in the context of each patient’s clinical presentation in warranted.

Shellfish allergies are up to seven times more prevalent in Asia compared to Western countries, and are the leading cause of anaphylaxis here [17]. We identified 14.3% of subjects with prawn sensitization on SPT, but only 1.5% when measured by ISAC. It is difficult to comment if the sensitization on SPT corresponded to clinical allergy as we did not collect data concerning post-exposure symptoms. Nevertheless, the agreement between the two methods for this allergen seemed poor. We explored whether the disproportionately high frequency of sensitization on SPT was attributable to cross-reactivity with the ubiquitous HDM tropomyosin [21,22]. However, sensitization to HDM tropomyosin (Der p 10) was infrequent, both on ISAC and ImmunoCAP. In addition, we found little association between tropomyosin sensitization (as measured by ImmunoCAP) with shellfish sensitization or, in fact, with HDM sensitization.

The low rates of sensitization to grass and tree pollen is known in our tropical environment, where plants are less reliant on pollination by air. Considering the humidity in our region, we expected a higher frequency of sensitization to mould. The low allergenic potential of mould allergens and the complexity of mould allergen isolation may have contributed to this observation [23]. Further studies are needed to understand mould sensitization and produce reliable mould antigens for in-vivo and in-vitro tests.

### Comparison between methods & ISAC’s role in Asia

A previous study of Korean patients with AD reported good correlation between ISAC and SPT. However, only a narrow range of allergens was included, the population was small and all subjects were of a single atopic phenotype, thus limiting the generalizability of the study’s conclusions [24]. In our current study, the agreement between SPT and ISAC was mostly slight to moderate. Greatest agreement between the two test methods was documented with the HDM (κ = 0.64). The agreement between ISAC and SPT for the remaining allergens was rather poor, although this analysis was constrained by the relatively low rates of sensitization to allergens other than HDM. The low rate of sensitization to the bulk of the 112 allergens in the ISAC panel underscores the limited utility of ISAC in Asia. Besides, agreement was suboptimal even for the most prevalent allergen.

This is the first Asian study attempting to examine the applicability of multiplex molecular allergy diagnostic methods in a mixed group of atopic and non-atopic individuals. The inclusion of non-atopics improves the validity of our results. In addition, we collected a large amount of data to characterize our subjects’ atopic symptoms to determine the clinical significance of sensitization. Nevertheless, there were several limitations. The definitions of atopic phenotypes were based on self-reported symptoms of disease. However, we attempted to reduce reporting bias through the use of validated surveys. The use of questionnaires to characterize exposure based symptoms would have been helpful to define the clinical relevance of sensitization to certain allergens such as food or latex. Future studies with a larger number of patients and specific exposure based symptoms may help to carve out a potential role for multiplex assays.

In conclusion, in Asia, the utility of ISAC in its current make-up is limited by various factors, including differences in the prevalence of atopy (which may be genetically determined) and environmental make-up. With its low cost and rapid turnover, SPTs maintain their central role in allergy diagnostics. ISAC should not be used as a screening tool, but has a role in directing immunotherapy targets and discerning primary from cross reacting allergens in polysensitized individuals. As locally relevant allergen species may differ, the allergenic sources in the test panel should mirror what is locally prevalent, so that results of the test have improved clinically relevance. It may be worthwhile to adjust the antigen spectrum on the ISAC panel to regional differences, in order to increase its applicability locally.

## Additional file

Additional file 1: Table S1. Agreement between Skin Prick Test (SPT) and ISAC.
