# Supplementary material for: Evaluation of the applicability of the Immuno-solid-phase allergen chip (ISAC) assay in atopic patients in Singapore
Source: Clin Transl Allergy. 2015 Feb 27;5:9. doi: 10.1186/s13601-015-0053-z (PMC4349609; doi:10.1186/s13601-015-0053-z)
Supplement: Additional file 1: Table S1. — Agreement between Skin Prick Test (SPT) and ISAC. [file 13601_2015_53_MOESM1_ESM.docx]

**Additional file 1: Table S1**

Agreement between Skin Prick Test (SPT) and ISAC

| **SPT Allergen** | **ISAC allergen** | **Agreement**  **Kappa (95%CI)** | **p** |
| --- | --- | --- | --- |
| *Alternaria tenuis* | Alt a 1 | * |  |
|  | Alt a 6 | * |  |
| *Cladosporium herbarum* | Cla h 8 | * |  |
| *Aspergillus fumigatus* | Asp f 1 | * |  |
|  | Asp f 3 | -0.02 (-0.04 to 0.01) | 0.877 |
|  | Asp f 6 | -0.03 (-0.06 to -0.002) | 0.786 |
| *Penicillium notatum* | N.A. | ᶱ |  |
| *Dermatophagoides farinae* | Der f 1 | 0.46 (0.30-0.63) | < 0.001 |
|  | Der f 2 | 0.55 (0.39-0.71) | < 0.001 |
|  | Der f (total) | 0.63 (0.46-0.79) | < 0.001 |
| *Dermatophagoides pteronyssinus* | Der p 1 | 0.43 (0.26-0.59) | < 0.001 |
|  | Der p 2 | 0.49 (0.33-0.65) | < 0.001 |
|  | Der p 10 | 0.04 (-0.05-0.08) | 0.186 |
|  | Der p (total) | 0.56 (0.40-0.73) | < 0.001 |
| *Blomia tropicalis* | Blo t 5 | 0.60 (0.44-0.77) | < 0.001 |
| Cockcroach | Bla g 1 | * |  |
|  | Bla g 2 | * |  |
|  | Bla g 5 | * |  |
|  | Bla g 7 | * |  |
|  | Bla g (total) | * |  |
| Grass | Amb a 1 | * |  |
|  | Art v 1 | * |  |
|  | Art v 3 | * |  |
|  | Che a 1 | -0.02 (-0.06-0.02) | 0.685 |
|  | Cyn d 1 | 0.26 (-0.03-0.55) | < 0.001 |
|  | Par j 2 | * |  |
|  | Phl p 1 | 0.33 (0.03-0.63) | < 0.001 |
|  | Phl p 11 | * |  |
|  | Phl p 12 | * |  |
|  | Phl p 2 | 0.14 (-0.1-0.38) | 0.013 |
|  | Phl p 4 | * |  |
|  | Phl p 5 | 0.22 (-0.06-0.51) | 0.007 |
|  | Phl p 6 | 0.14 (-0.10-0.38) | 0.013 |
|  | Phl p 7 | * |  |
|  | Pla l 1 | * |  |
|  | Sal k 1 | * |  |
|  | Grasses (total) | 0.30 (-0.002-0.59) | 0.002 |
| Cereal | Fag e 2 | * |  |
|  | Tri a 14 | * |  |
|  | Tri a 19.0101 | * |  |
|  | Tri a aA_TI | * |  |
|  | Cereals (total) | * |  |
| Herb | - | ᶱ |  |
| Tree mix 1 and  Tree mix2 | Aln g 1 | * |  |
|  | Bet v 1 | 0.28 (-0.03-0.59) | < 0.001 |
|  | Bet v 2 | * |  |
|  | Bet v 4 | * |  |
|  | Cry j 1 | * |  |
|  | Cup a 1 | * |  |
|  | Ole e 1 | * |  |
|  | Ole e 7 | * |  |
|  | Ole e 9 | * |  |
|  | Pla a 1 | * |  |
|  | Pla a 2 | * |  |
|  | Pla a 3 | * |  |
|  | Trees (total) | 0.28 (-0.03-0.59) | < 0.001 |
| Latex | Hev b 1 | -0.02 (-0.05-0.01) | 0.824 |
|  | Hev b 3 | -0.02 (-0.05-0.01) | 0.824 |
|  | Hev b 5 | * |  |
|  | Hev b 6.01 | * |  |
|  | Hev b 8 | -0.02 (-0.05-0.01) | 0.824 |
|  | Latex (total) | -0.02 (-0.05-0.01) | 0.824 |
| Dog | Can f 1 | 0.15 (-0.03-0.32) | 0.032 |
|  | Can f 2 | 0.06 (-0.05-0.17) | 0.106 |
|  | Can f 3 | 0.06 (-0.05-0.17) | 0.106 |
|  | Can f 5 | 0.12 (-0.03-0.26) | 0.022 |
|  | Can f (total) | 0.25 (0.05-0.45) | 0.002 |
| Cat | Fel d 1 | 0.48 (0.24-0.71) | < 0.001 |
|  | Fel d 2 | * |  |
|  | Fel d 4 | 0.13 (-0.07-0.32) | 0.058 |
|  | Fel d (total) | 0.45 (0.21-0.68) | < 0.001 |
| Prawn | Pen m 1 | * |  |
|  | Pen m 2 | -0.04 (-0.12-0.03) | 0.680 |
|  | Pen m 4 | * |  |
|  | Pen m (total) | -0.04 (-0.12-0.03) | 0.680 |
| Curry | - | ᶱ |  |
| Coffee | - | ᶱ |  |
| Wheat | Fag e 2 | * |  |
|  | Tri a 14 | * |  |
|  | Tri a 19.0101 | * |  |
|  | Tri aaA_TI | * |  |
|  | Wheat (total) | * |  |
| Soya | Gly m 4 | * |  |
|  | Gly m 5 | * |  |
|  | Gly m 6 | * |  |
|  | Gly m (total) | * |  |
| Pork | - | ᶱ |  |

* Kappa not calculated due to absent / low number of sensitized individuals picked up by ISAC

ᶱ Kappa not calculated due to absence of corresponding allergen within ISAC panel
